# Disentangling the within- and between-person effects of personality on income for men and women

**DOI:** 10.1098/rsos.231750

**Published:** 2024-05-22

**Authors:** Simon Kemp, Kumar Yogeeswaran, Samantha Stronge, Mona Yaghoubi, Chris G. Sibley

**Affiliations:** ^1^ School of Psychology, Speech and Hearing, University of Canterbury, Christchurch, New Zealand; ^2^ Department of Economics and Finance, University of Canterbury, Christchurch, New Zealand; ^3^ School of Psychology, University of Auckland, Auckland, New Zealand

**Keywords:** income, personality, random-intercepts cross-lagged panel model, within- and between-person effects

## Abstract

Understanding the relationship between personality and income is a topic of interest across multiple disciplines. Correlations between people’s personalities and their incomes may arise because differences in stable personalities relate to income differences (between-person effects) or because changes in personality or income are later reflected in the other variable (within-person effects). The current research uses random-intercept cross-lagged panel models to disentangle the two sorts of effects to better understand the relationship between the six factors of personality and income. Using data from 6824 working-age adults in New Zealand across 4 years, we found between-person effects showing higher incomes were obtained by both men and women who were more extraverted, agreeable and open, and less neurotic. Within-person effects showed that earning a higher income was associated with higher neuroticism and lower extraversion over time, while higher extraversion was associated with a lower income over time.

## 1. Personality and income

A large amount of research has already sought to link personality factors to other variables, including income. Personality refers to individual differences in how people think, feel and behave and represents a foundational aspect of human psychology (e.g. [1]). It has often been measured using the Big-Five personality structure, which considers five personality traits: openness, conscientiousness, extraversion (as opposed to introversion), agreeableness, and neuroticism (as opposed to emotional stability). The model is well validated (e.g. [2]). Recently, some researchers have supplemented the Big-Five personality traits with a sixth personality variable: Honesty–humility [3,4], in part because honesty–humility is seen as opposed to the dark triad of narcissism, Machiavellianism and psychopathology (e.g. [5]).

Income is a relatively objective measure and measured quite differently to personality variables. Perhaps most basically, both personality and income are considered important. Personality is an important variable in distinguishing people’s psyches; income is regarded as very significant for an individual’s welfare.

The links between personality factors and work-related variables have been studied often enough for quantitative analyses of the meta-analyses of these relationships to be undertaken for conscientiousness [6], agreeableness [7] and extraversion [8]. These analyses confirm a large number of relationships. For the present, it is worth noting that they showed agreeableness was weakly associated with lower salaries over 6 studies [7] and extraversion weakly associated with higher salaries over 15 studies [8].

More recently, Alderotti *et al.* [9] produced a meta-analysis of 62 articles investigating the relationship between the Big-Five personality traits and personal earnings. Overall, earnings had positive relationships with openness, conscientiousness, and extraversion, but negative relationships with agreeableness and neuroticism. However, although these associations were statistically significant, they were weak. Moreover, both the strength and direction of the effects were very variable, with all personality factors showing both positive and negative effects depending on the particular study.

Such findings indicate that it is important to look closely at possible moderators of the effects. Alderotti *et al.* [9] identified significant moderator effects of culture (anglophone or not), disciple journal and gender, although not of the time of the study or the number of items used in the scales. Studies relating personality factors to income differ in other ways besides those examined by Alderotti *et al.* [9]. For example, some studies (e.g. [10, 11]) have used raw or linearly scaled income measures. Others (e.g. [12–15]) used logarithmically scaled income measures. Some previous studies have measured income and personality factors at the same time (e.g. [11,13,14,16]), while in others, the personality factors were measured some items before income (e.g. [10,12,15]). This difference is important if, as in the present study, one wishes to infer the direction of the effect.

Measuring personality factors before measuring income implies that the researcher is interested in the effect of personality on income rather than the other way around. As personality is generally thought to be fairly stable while people’s income can show a great deal of variability over time, this focus makes sense. However, there is evidence that personality is not completely fixed and that causation may occasionally flow in the opposite direction. People are often motivated to change their personality [4]. For example, they might like to become more conscientious. Socioeconomic differences have been shown to affect brain structure and immune system functioning [17,18], and presumably personality too. Of direct relevance to the present study, Wu [19] found among 1814 Australian employees that an increase in time demand at work produced increased neuroticism and decreased extraversion and conscientiousness over time, while an increase in job control produced increases in agreeableness, conscientiousness and openness. Hirschi *et al*. [20] investigated 4767 German SOEP respondents using cross-lagged panel analyses and found that higher incomes subsequently produced decreases in neuroticism and increases in openness. Overall, then, we should not be surprised to find both causal directions at work—personality to income and income to personality—although not necessarily to the same extent.

While previous research on the topic has been valuable, one limitation is that it does not separate between- and within-person effects. Between-person effects in the present context imply fundamentally that personality traits are stable, and these have a consistent effect on people’s incomes. (This does not imply people’s incomes will remain stable because there are a host of other variables affecting income.) Within-person effects, depending on which effects are found, might imply that one’s personality traits are affected by one’s income, as well as by other variables, and these personality changes might in turn affect one’s subsequent income. The current research makes an important contribution to the literature by separating the between-person, trait-like, time-invariant component of the relationship between personality and income from the within-person, state-like and time-variant component of the relationship [21].

## 2. Gender differences in personality and income relationships

The work experience of men and women is often different. For example, in many countries, women receive, on average, lower work-related incomes than men, for the most part because they tend to be employed in different occupations (e.g., [22]). Given this difference, it is reasonable to ask if the relationship between personality and income also differs for men and women. Alderotti *et al.*’s [9] meta-analysis found some gender moderation effects: both the positive effect of openness on earnings and the negative effect of neuroticism were stronger for women. Nyhus and Pons [13], for example, found that although the direction of the effects was similar, overall personality effects accounted for a higher percentage of the variance in income for women (5%) than men (0.7%). Mueller and Plug [12] also found income to be more related to personality among women than men, although there was a smaller difference in R^2^ (women: 6%; men: 5%). Judge *et al*. [10] focused particularly on whether being disagreeable financially paid off more for men than women. In two studies (1 and 3), they found this effect, but it did not appear in Study 2. It is possible that this difference in results reflects differences in the samples as Study 2 surveyed 25–74 year-olds, while Study 1 used a broad cohort study of people aged 23–27 and the respondents of Study 3 were in their 50s.

Judge *et al.*’s [10] Study 2 is an example of the very much broader result that gender differences that have often been expected, even touted, in different areas do not always replicate. Hyde [23] reviewed a large number of areas—for example, mathematical abilities—in which gender similarities appear more frequently than gender differences, and, where gender differences have turned up, they are often small. Indeed, Hyde ([24], p. 581) presents a strong case, based on reviewing 46 meta-analyses, that ‘males and females are similar on most, but not all, psychological variables’.

Putting the different results together, there may be an overall tendency for personality to be more related to women’s incomes, but the emergence and size of the difference probably depend on other unknown factors. As Hyde [23] points out, one should not expect such a tendency to emerge in all societies or sub-groups of societies.

## 3. Current research

The present study aimed to broaden the investigation of the relationship between personality and income in three important ways. First, and most importantly, the current research uses panel data following participants across multiple waves, allowing us to measure both the effects of personality on income and those of income on personality. In doing this, we decided to make use of a new method for analysing cross-lagged panels put forward by Hamaker *et al*. [21]. There has recently been criticism of conventional cross-lagged panel models (CLPMs), in part because they tend to treat between- and within-subject effects similarly, although the causal influences that might be at work can be very different (e.g. [25,26]). Hamaker *et al*.’s [21] random-intercept cross-lagged panel model (RI-CLPM) provides a way of separating the between-subject relationships between two sets of variables from their within-subject relationships. In the present context, this means separating the stable trait-like relationships between personality and income from the changes in individual personality that might produce changes in income. Further details of the method are given below. For the moment, it is important to note that this novel method provides more detail about the personality–income relationships than what has been explored in previous work. Second, the current research expands previous work using the Big-Five personality traits to a Big-Six model that includes honesty–humility. Third, the current research advances our understanding of both the within-person and between-person effects of the relationship between personality and income by considering the relationship separately for men and women.

The current research addresses these goals using 4 years of longitudinal data from a nationally representative sample of working adults in New Zealand. New Zealand is a small country in the South Pacific with much cultural similarity to other predominantly Anglophone countries such as the United Kingdom, United States, Canada and Australia (e.g. [27]). On the other hand, compared to these countries, New Zealand firm sizes are often smaller [28], and it is quite conceivable that this difference might matter for personality–income relationships (a point we will further consider in §6).

**Figure 1.**
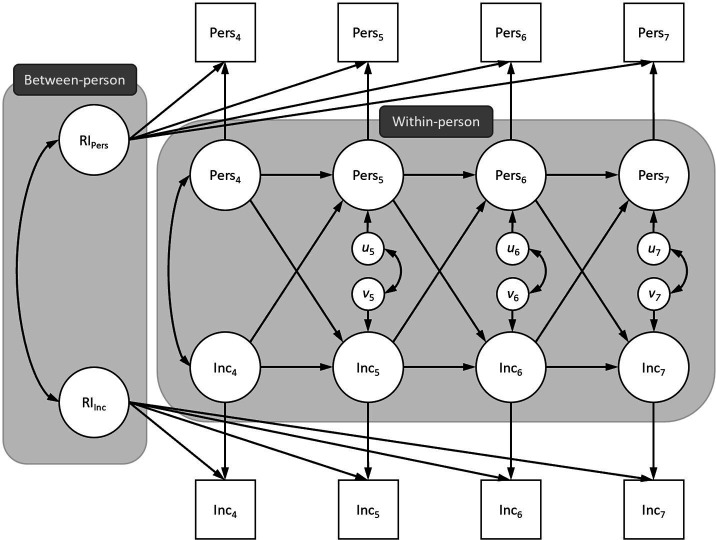
Representation of a RI-CLPM investigating the association between personality and income across four waves of data. *Note:* A separate model following this pattern was run for each personality trait.

The RI-CLPM model (see figure 1 below) effectively provides a formal test of the relationship between personality and income. For each personality variable, the model investigates whether there is a between-subjects influence of that variable on income, and also two within-subject effects: Do changes in that personality variable affect income, and do changes in income affect that personality variable? The model also measures the effects of personality on subsequent personality levels and the effects of income on subsequent income levels, although these were not of much interest to us.

Beyond these research goals mentioned above, we did not formulate specific hypotheses, in part, because a very clear feature of previous research has been that the ‘usual’ findings, for example, that disagreeable people earn more, do not always hold true. Given this evidence of variability, and considering our study’s differences from previous research (especially in analytical method and population examined), our work can be considered exploratory.

## 4. Method

### 4.1. Sampling procedure

The New Zealand Attitudes and Values Study (NZAVS) is a longitudinal panel study of New Zealand adults. Participants are sampled from the New Zealand electoral roll, which is publicly available for scientific research and represents all citizens over 18 years of age who were eligible to vote (regardless of whether they choose to vote). Online and postal questionnaires are sent to all active participants annually. The current research uses data from all of the time points where personal income was measured; Time 4 (2012; *n* = 12 179), Time 5 (2013; *n* = 18 261), Time 6 (2014; *n* = 15 820) and Time 7 (2015; *n* = 13 942) of the NZAVS.

Booster sampling was conducted at Time 4 (44% of participants were new to the study at this time point) and Time 5 (42% of participants were new to the study at this time point) to boost sample size and increase sample diversity, with participants randomly selected from the New Zealand electoral roll. No booster sampling was conducted at Time 6 and Time 7. Year-to-year retention was high for all time points: Time 4 (83.7% retained from Time 3), Time 5 (80.8% retained from Time 4), Time 6 (81.5% retained from Time 5) and Time 7 (79.3% retained from Time 6). For full details regarding sampling and procedure, refer to Sibley [29]. Informed consent was obtained from all participants for each wave of the study.

### 4.2. Participants

Participants were restricted to those who responded to at least two of the four time points, were between the ages of 18 and 65 years (i.e. the working age population in New Zealand, although there is no requirement to retire at age 65) at the first time point they responded, and were employed in full-time work (defined as 30 or more self-reported hours working in paid employment) to be included in the analyses. In total, 6824 participants were included. (Means and standard deviations and bivariate correlations for all variables are available in the electronic supplementary material, table S1 and comparisons of mean income across age categories are in electronic supplementary material, table S2.)

### 4.3. Measures

All measures were embedded within the larger NZAVS questionnaire. For a full list of measures, refer to Sibley [29].

#### 4.3.1. Personal income

Income was measured with an open-ended question that read, ‘Please estimate your own personal earnings (before tax) for the year 2013 [2014/2015]’. Income was log-transformed (natural logarithms) for use in the analysis.

#### 4.3.2. Personality

Big-Five personality was assessed using the Mini-IPIP6 (International Personality Item Pool), which adapted items from the Mini-IPIP [30]. All traits are measured with four items on a scale from 1 (very inaccurate description of me) to 7 (very accurate). Extraversion was assessed with: ‘[I] am the life of the party’, ‘[I] don’t talk a lot’ (reverse-scored), ‘[I] keep in the background’ (reverse-scored) and ‘[I] talk to a lot of different people at parties’ (*α*’s = 0.75–0.77). Agreeableness was assessed with: ‘[I] sympathize with others’ feelings’, ‘[I] am not interested in other people’s problems’ (reverse-scored), ‘[I] feel other’s emotions’ and ‘[I] am not really interested in others’ (reverse-scored; *α*’s = 0.72–0.74). Conscientiousness was assessed with: ‘[I] get chores done right away’, ‘[I] like order’, ‘[I] make a mess of things’ (reverse-scored) and ‘[I] often forget to put things back in their proper place’ (reverse-scored; *α*’s = 0.65–0.69). Neuroticism was assessed with: ‘[I] have frequent mood swings’, ‘[I] am relaxed most of the time’ (reverse-scored), ‘[I] get upset easily’ and ‘[I] seldom feel blue’ (reverse-scored) (*α*’s = 0.69–0.73). Openness to experience was assessed with: ‘[I] have a vivid imagination’, ‘[I] have difficulty understanding abstract ideas’ (reverse-scored), ‘[I] do not have a good imagination’ (reverse-scored) and ‘[I] am not interested in abstract ideas’ (reverse-scored; *α*’s = 0.70–0.73).

Honesty–humility was also assessed with four items. Two items were adapted from the HEXACO Honesty-Humility Scale [3]: ‘[I] would like to be seen driving around in a very expensive car’, and ‘[I] would get a lot of pleasure from owning expensive luxury goods’ and the remaining two items were adapted from the Psychological Entitlement Scale [31]: ‘[I] feel entitled to more of everything’, ‘[I] deserve more things in life’ (all items reverse-coded; *α*’s = 0.75–0.77). These four items have been validated for use in the New Zealand context as markers of a distinct ‘sixth’ factor in models including the Big-Five [30,32].

### 4.4. Analytic plan

We investigated longitudinal change using RI-CLPM [21]. We estimated a separate RI-CLPM between income and each personality trait using *Mplus* v. 8.4 and maximum likelihood with robust estimation of standard errors. We used full information maximum likelihood estimates allowing us to use data from participants who only took part in some time points and estimated bias-corrected 95% confidence intervals using 5000 bootstrapped resamples (with replacement) for all pathways. We focus on unstandardized coefficients as standardization is more complicated in a multi-level model than in ordinary least-squares regression.

A RI-CLPM builds upon a standard CLPM while addressing some of its shortcomings. It has been argued that standard cross-lagged models combine between-person stability and within-person change within a single estimate [21,25]. Therefore, results from a CLPM may not represent actual change over time, particularly when the variables being studied are of a stable trait-like nature-like personality. This also means that CLPM assumes each individual varies around a group mean with no enduring individual differences over time, which is not a good model for personality traits [21]. Simulated data and empirical illustrations have shown that, in these circumstances, a CLPM can produce biased estimates in terms of the strength, direction and presence of an effect [21,25,26]. In contrast, a RI-CLPM breaks down observed variables into two components: (i) the between-person, trait-like, time-invariant component, and (ii) the within-person, state-like, time-varying component [21]. This allows trait-like stability at the between-person level to be adjusted so that change over time at the within-person level can be isolated.

Figure 1 represents the RI-CLPM model used for all six personality traits [21,33]. Beginning with the between-person component, we created a mean of the four observed personality variables that were measured at each time point, before estimating a random intercept consisting of the four means with factor loadings fixed to 1. A similar random intercept was estimated for income using the four observed income variables with factor loadings fixed to 1. We allowed the two random intercepts for personality and income to correlate. These random intercepts represent an average score of each participant’s income and personality for the entire 4 year period of measurement. Their inclusion in the model allows each person their own individual mean for personality and income that they vary around over time (rather than the group mean allowed in the CLPM) and accounts for the stability of between-person differences over time [21].

After adjusting for between-person stability, what remains is within-person change over time. The within-person component represents deviations away from the individual mean at each time point [21]. Even with very stable traits like personality, we do not expect people to score identically each year. Instead, a person’s actual scores will fluctuate above or below their average score over time. To model this, a latent variable was estimated for personality (using the observed mean) and income at each individual time point. The residual variance of the observed personality mean variables and observed income variables were constrained to 0, so all variance was forced into either the between-person random intercepts or into the within-person latent variables. The within-person latent variables were then used to estimate the cross-lagged and auto-regressive associations; Time 7 personality and income were regressed onto Time 6 personality and income, Time 6 personality and income was regressed onto Time 5 personality and income, which was in turn regressed onto Time 4 personality and income. We constrained the pathways to equality across time point pairs, estimating a stationary process, as there was no theoretical basis to expect the effects to differ from one year to the next.

To account for any potential measurement error unique to a specific time point, contemporaneous covariances were estimated between the personality and income within-person latent variables. Because we assume a constant process rather than a process that begins at Time 4, the correlations between the random intercepts and within-person latent variables at Time 4 were constrained to 0 [21]. Note that adjusting for the between-person component means that all time-invariant differences between people in the within-person component such as sex, ethnicity or date of birth are controlled automatically [26].

## 5. Results

The cross-lagged within-person effects examine whether deviations at one time-point predict deviations away from the average score of *other* variables in future time points (while controlling for any past deviations in the other variables [21]). Significant cross-lagged associations were found between income and both extraversion and neuroticism (see tables 1 and 2). The results showed that an increase in extraversion at one time-point was associated with a *decrease* in income at the next time point. Similarly, an increase in income at one time-point was associated with later decreases in extraversion, as well as later increases in neuroticism. Cross-lagged (within-subject) pathways were non-significant for all other traits. For conscientiousness and openness, this meant the association between personality and income was only significant in the between-person component. People who were more conscientious or more open also tended to make more money; however, causal relationships could not be established between these personality traits and income. For agreeableness and honesty–humility, personality and income were simply not significantly associated at any level.

**Table 1.** Model fit statistics for separate RI-CLPMs examining the association between each personality trait and personal income for the overall model, women and men.

	extraversion	agreeableness	conscientiousness	neuroticism	openness	honesty–humility
*overall*						
*χ* ^2^_(df)_	28.67_(17)_*	48.46_(17)_***	34.52_(17)_**	52.50_(17)_***	42.64_(17)_***	32.37_(17)_*
CFI	0.999	0.998	0.999	0.998	.0998	0.999
RMSEA [95% CI]	0.010 [0.002, 0.016]	0.016 [0.011, 0.022]	0.012 [0.006, 0.018]	0.017 [0.012, 0.023]	0.015 [0.009, 0.021]	0.012 [0.005, 0.017]
SRMR	0.032	0.038	0.034	0.035	0.040	0.035
*women*						
*χ* ^2^_(df)_	20.56_(17)_	35.10_(17)_**	34.16_(17)_**	37.07_(17)_**	31.72_(17)_*	35.61_(17)_**
CFI	1.000	0.997	0.998	0.997	0.998	0.998
RMSEA [95% CI]	0.008 [0.000, 0.018]	0.017 [0.009, 0.025]	0.017 [0.008, 0.025]	0.018 [0.010, 0.026]	0.016 [0.007, 0.024]	0.018 [0.009, 0.026]
SRMR	0.035	0.048	0.040	0.041	0.044	0.043
*men*						
*χ* ^2^_(df)_	35.98_(17)_**	36.35_(17)_**	37.90_(17)_**	60.83_(17)_***	53.00_(17)_***	35.74_(17)_**
CFI	0.998	0.997	0.997	0.994	0.995	0.998
RMSEA [95% CI]	0.019 [0.010, 0.027]	0.019 [0.010, 0.027]	0.019 [0.011, 0.028]	0.028 [0.021, 0.036]	0.025 [0.018, 0.033]	0.018 [0.010, 0.027]
SRMR	0.035	0.038	0.037	0.040	0.043	0.036

*p < 0.05, **p < 0.01, ***p < 0.001

CFI, Comparative fit index; RMSEA, Root mean square error of approximation; SRMR, Standardized root mean square residual.

**Table 2.** RI-CLPM results for the between-person component and the auto-regressive and cross-lagged within-person components for all personality traits and income.

	extraversion			agreeableness			conscientiousness		
	*b*	*95% CI*	*p*	*b*	*95% CI*	*p*	*b*	*95% CI*	*p*
*between-person correlation*									
personality RI ↔ income RI	0.048***	[0.027, 0.070]	<0.001	0.006	[−0.010, 0.025]	0.479	0.028**	[0.009, 0.048]	0.005
*auto-regressive paths*									
personality → personality	0.076**	[0.032, 0.118]	0.001	0.038	[−0.003, 0.081]	0.083	0.068**	[0.026, 0.112]	0.002
income → income	0.057	[−0.105, 0.253]	0.531	0.058	[−0.105, 0.251]	0.526	0.056	[−0.106, 0.250]	0.536
*cross-lagged paths*									
personality → income	−0.058**	[−0.104, −0.018]	0.008	−0.020	[−0.064, 0.022]	0.362	−0.014	[−0.071, 0.038]	0.622
income → personality	−0.028**	[−0.048, −0.010]	0.003	−0.011	[−0.038, 0.014]	0.398	−0.006	[−0.040, 0.024]	0.735
	neuroticism			openness			honesty-humility		
	*b*	*95% CI*	*p*	*b*	*95% CI*	*p*	*b*	*95% CI*	*p*
*between-person correlation*									
personality RI ↔ income RI	−0.103***	[−0.127, −0.083]	<0.001	0.065***	[0.044, 0.087]	<0.001	0.002	[−0.019, 0.026]	0.835
*auto-regressive paths*									
personality → personality	0.106***	[0.063, 0.147]	<0.001	0.052*	[0.010, 0.098]	0.020	0.137***	[0.096, 0.179]	<0.001
income → income	0.059	[−0.102, 0.253]	0.518	0.057	[−0.106, 0.253]	0.531	0.056	[−0.108, 0.248]	0.541
*cross-lagged paths*									
personality → income	0.023	[−0.014, 0.066]	0.251	0.002	[−0.046, 0.047]	0.944	−0.011	[−0.049, 0.025]	0.575
income → personality	0.039**	[0.012, 0.070]	0.007	−0.027	[−0.061, 0.004]	0.103	0.006	[−0.020, 0.033]	0.642

*p < 0.05, **p < 0.01, ***p < 0.001

To summarize, across 4 years, the RI-CLPM showed that people with higher incomes also tended to be people who were more extraverted, more conscientious, more open to experience and less neurotic. However, when income increased, it was associated with decreases in extraversion and increases in neuroticism. Likewise, if extraversion increased it was associated with a reduced income.

### 5.1. Gender analyses

We further investigated how the longitudinal associations between personality and income might differ for men and women. We used the same set of RI-CLPM analyses as described above, but estimated the models separately for men and women. Model fit was good for all personality traits for women and men (see table 1). Unstandardized results for the RI-CLPMs are presented in table 3 for women and table 4 for men. Bivariate correlations and descriptive statistics for men and women are presented in electronic supplementary material, tables S3 and S4.

**Table 3.** RI-CLPM results for the between-person component and the auto-regressive and cross-lagged within-person components for all personality traits and income for women.

	extraversion			agreeableness			conscientiousness		
	*B*	*95% CI*	*p*	*b*	*95% CI*	*p*	*b*	*95% CI*	*p*
*between-person correlation*									
personality RI ↔ income RI	0.043**	[0.011, 0.076]	0.008	0.055***	[0.030, 0.087]	<0.001	0.034*	[0.003, 0.066]	0.030
*auto-regressive paths*									
personality → personality	0.059*	[0.001, 0.120]	0.049	0.036	[−0.021, 0.096]	0.227	0.073*	[0.012, 0.137]	0.020
income → income	0.106	[−0.109, 0.384]	0.399	0.106	[−0.107, 0.384]	0.397	0.105	[−0.108, 0.383]	0.404
*cross-lagged paths*									
personality → income	−0.044	[−0.115, 0.017]	0.194	−0.060	[−0.134, 0.003]	0.090	0.003	[−0.087, 0.081]	0.949
income → personality	−0.024	[−0.048, 0.001]	0.053	−0.014	[−0.045, 0.018]	0.381	0.001	[−0.043, 0.041]	0.952
	neuroticism			openness			honesty-humility		
	*b*	*95% CI*	*p*	*b*	*95% CI*	*p*	*b*	*95% CI*	*p*
*between-person correlation*									
personality RI ↔ income RI	−0.090***	[−0.124, −0.061]	<0.001	0.054**	[0.022, 0.085]	0.001	0.026	[−0.005, 0.063]	0.127
*auto-regressive paths*									
personality → personality	0.120***	[0.065, 0.177]	<0.001	0.059	[−0.003, 0.122]	0.066	0.119***	[0.060, 0.182]	<0.001
income → income	0.104	[−0.109, 0.370]	0.398	0.101	[−0.109, 0.376]	0.411	0.101	[−0.112, 0.378]	0.413
*cross-lagged paths*									
personality → income	0.019	[−0.038, 0.086]	0.540	0.027	[−0.047, 0.095]	0.452	−0.042	[−0.106, 0.009]	0.147
income → personality	0.038*	[0.004, 0.075]	0.033	−0.035	[−0.074, 0.000]	0.063	0.013	[−0.023, 0.045]	0.456

*p < 0.05, **p < 0.01, ***p < 0.001

**Table 4.** RI-CLPM results for the between-person component and the auto-regressive and cross-lagged within-person components for all personality traits and income for men.

	extraversion			agreeableness			conscientiousness		
	*B*	*95% CI*	*p*	*b*	*95% CI*	*p*	*b*	*95% CI*	*p*
*between-person correlation*									
personality RI ↔ income RI	0.071***	[0.044, 0.099]	<0.001	0.036**	[0.015, 0.056]	0.001	0.051***	[0.029, 0.077]	<0.001
*auto-regressive paths*									
personality → personality	0.094**	[0.031, 0.161]	0.004	0.037	[−0.017, 0.097]	0.202	0.060	[−0.001, 0.124]	0.057
income → income	−0.025	[−0.404, 0.247]	0.869	−0.026	[−0.408, 0.246]	0.867	−0.029	[−0.412, 0.242]	0.850
*cross-lagged paths*									
personality → income	−0.069*	[−0.128, −0.014]	0.019	0.021	[−0.025, 0.076]	0.401	−0.036	[−0.114, 0.024]	0.295
income → personality	−0.030	[−0.063, 0.000]	0.062	−0.006	[−0.052, 0.036]	0.793	−0.015	[−0.069, 0.030]	0.542

	neuroticism			openness			honesty-humility		
	*b*	*95% CI*	*p*	*b*	*95% CI*	*p*	*b*	*95% CI*	*p*
*between-person correlation*									
personality RI ↔ income RI	−0.071***	[−0.102, −0.046]	<0.001	0.053***	[0.029, 0.077]	<0.001	0.019	[−0.008, 0.050]	0.191
*auto-regressive paths*									
personality → personality	0.087*	[0.022, 0.157]	0.011	0.047	[−0.013, 0.109]	0.138	0.157***	[0.097, 0.219]	<0.001
income → income	−0.024	[−0.405, 0.251]	0.876	−0.030	[−0.417, 0.243]	0.846	−0.026	[−0.416, 0.245]	0.864
*cross-lagged paths*									
personality → income	0.036	[−0.004, 0.088]	0.114	−0.020	[−0.073, 0.038]	0.465	0.020	[−0.027, 0.069]	0.411
income → personality	0.034	[−0.005, 0.094]	0.160	−0.001	[−0.055, 0.064]	0.979	−0.006	[−0.052, 0.043]	0.795

*p < 0.05, **p < 0.01, ***p < 0.001

Beginning with the between-person component for women (*n* = 3555), the correlations between the random intercepts were significant and positive for extraversion, agreeableness, conscientiousness and openness, and significant and negative for neuroticism. The auto-regressive within-person effects for women showed significant and positive associations for extraversion, conscientiousness, neuroticism and honesty–humility, indicating that changes in these traits at one time-point would persist into future time points. The cross-lagged within-person effects for women showed just one significant effect; an increase in income at one time-point was associated with higher neuroticism at later time points.

For men (*n* = 3261), the correlations between the random intercepts were significant and positive for extraversion, agreeableness, conscientiousness and openness, and negative for neuroticism. Therefore, the pattern of significant between-person effects does not differ between men and women. The auto-regressive within-person effects showed positive associations for extraversion, neuroticism and honesty–humility; unlike for women, there was no significant auto-regressive association for conscientiousness for men. Finally, one cross-lagged within-person effect was significant and negative for men; an increase in extraversion at one time-point was associated with a *lower* income later.

To summarize, higher income preceded higher neuroticism for women, while higher extraversion preceded a lower income for men. However, it is important to note that the associations were not necessarily different between men and women. The confidence interval for the income to neuroticism association found for women overlaps with the confidence interval of the same association for men, and the effect sizes are similar and are in the same direction. The same is true for the extraversion to income association among men. The differences in significant findings may simply reflect the reduced sample size in these subsample analyses relative to the overall analysis. Similarly, the cross-lagged effect found in the overall model where higher income preceded lower extraversion was not present for either gender (the association was approaching significance and in the same direction for both men and women). In short, gender does not appear to strongly moderate the associations between personality and income over time.

## 6. Discussion

The current research used a novel approach of random intercept CLPMs to examine both between-person *and* within-person changes between the Big-Six personality variables (openness, conscientiousness, extraversion, agreeableness, neuroticism, and honesty–humility) and personal income using 4 years of longitudinal data from a nationally representative sample of New Zealand working-age adults. Additionally, the current work tested if the relationship between personality and income was similar or different among men and women by examining the between- and within-person effects separately.

The data revealed several interesting and important findings about the relationship between personality and income. First, extraversion predicts higher income within the overall population suggesting that more extraversion predicts higher income between-persons. This higher income could result from extraverts taking on leadership roles and high-paying jobs. Judge *et al*. [34] meta-analysed 222 correlations from 73 samples and concluded that extraversion was the most consistent correlate of leadership across study settings and leadership criteria. Using their findings, Green *et al*. [35] show that extraverted CEOs and CFOs earn 6–9% higher salaries, and that extraverted CFOs are more likely to be promoted to CEO. In addition, our findings are consistent with Bowles *et al*. [36] incentive-enhancing preferences theory and personality and performance literature. Bowles *et al*. [36] use a simple agent-principal model to propose how an employee’s personality traits interact with the employer’s incentivizing strategies. Offering the same incentive to the employees with high incentive-enhancing preferences (‘good worker’) induces stronger responses (i.e. working harder) compared to employees with low incentive-enhancing preferences (‘bad worker’). As a result, profit-maximizing employers recognize these preferences and pay more to the ‘good workers’. Applying this theoretical framework, Spurk and Abele [37] show a positive relationship between extraversion and salary, which is consistent with our findings. However, intriguingly, within-person increases in extraversion are related to decreases in income over time within the same individual, suggesting that while being more extraverted relates to higher income, becoming more extraverted over time only results in lower income.

Second, between-person increases in neuroticism were related to lower income. This finding is consistent with Bowles *et al*. [36] incentive-enhancing preferences theory, where individuals with neuroticism would be considered ‘bad workers’ and paid less. However, there were nuanced within-person effects of the same relationship. Specifically, an increase in income predicted increases in neuroticism during the following year.

Third, more agreeable, open, and conscientious people tend to earn more money. However, there is no within-person effect of agreeableness, openness, or conscientiousness on income suggesting that while being agreeable, open, and conscientious is generally related to more income, if the same person becomes more agreeable, open, or conscientious over time, it does not lead to greater income. Similarly, being honest and humble relates to greater income between-person, but if the same individual shows more honesty–humility over time, this does not translate into higher income. Nor do increases in income produce more agreeableness, openness, conscientiousness, or honesty–humility.

A number of features of our results resemble those from previous work. Note, firstly, that the associations between personality traits and income are generally small, both here and previously. Secondly, the previous finding that more neurotic people tend to have lower incomes was replicated here. On the other hand, we did not find that less agreeable and extraverted people had higher incomes. As previous research has relied on cross-sectional data or relied on statistical approaches that did not tease apart between- and within-person changes in personality on income (and vice versa), these differences highlight the importance of separating out between- and within-person variance. Our findings suggest that results from previous research could be attributed to between-person differences in agreeableness and extraversion rather than actual change within people over time. Interestingly, our exploratory investigation of how gender may change the associations between personality and income over time found no strong gender differences (cf. [23]). While previous work has found that personality was more strongly associated with income for women, they also found that effects were in the same direction for men and women [12,13].

More broadly, our method of analysis provides extra information regarding the patterns of causation between personality and income. The relationships we uncovered between neuroticism and income are the most striking example of this. Overall, our findings indicate that the relationship between personality and income is quite complex with certain dimensions of personality being more related to personal income than others. But more importantly, our analytic approach sheds light on the nuanced ways in which differences on certain personality traits within the population relate to income as opposed to how changes in a certain personality trait or income within the same individual influence the other across time.

### 6.1. Limitations and future directions

The current research has a number of strengths including its use of 4 years of longitudinal data from a large nationally representative sample of working adults to tease apart within- and between-person effects for the relationship between personality and income, its focus on all six dimensions of personality and its exploration of effects for men and women separately. However, the present work also has limitations that warrant future attention.

The reported results are based on a large national sample of New Zealand adults, but it is unclear how these findings would replicate in other national contexts. Alderotti *et al*.’s meta-analysis showed that culture does matter. For example, ‘[a]greeableness is more likely to be a liability in the United Kingdom’s labor market rather than in Japan’s’ ([9], p. 10). Other studies indicate that cultural effects can be nuanced. Elleman *et al*. [38] found that, within individual states in the United States, the relationship between personality and income disparity was strengthened if the analysis was conducted at a more fine-grained (postcode) level rather than at a state-wide level. Matz and Gladstone ([39], Studies 6 and 7) found that, within the United Kingdom and the United States, different regions have different rates of insolvencies and agreeableness. As New Zealand is a small country with generally smaller firm sizes [28], it is unclear if similar research carried out in larger and more industrialized economies such as the United States or Europe would replicate the observed findings. Nevertheless, New Zealand has a similar economic model to many other advanced western economies with comparable metrics on gender equality and other indexes, so it may be that the observed findings would emerge elsewhere too. Future work should examine this possibility more closely.

Another limitation of the current research is that the results reported here are based on an annual survey where responses are measured about a year apart. It may be that the effect of income on personality is short-term and a lag of 1 year misses out changes to personality occurring within a few months of changes in income that we do not detect here, or changes to personality persist for a few months, but not for a full year. A similar concern has been raised in other longitudinal research examining the relationship between self-esteem and depressive symptoms [40]. However, it is difficult to know what the correct time frame would be for examining such associations. One could argue that changes to one’s personality or income are usually slow and measuring these within a shorter duration would be inappropriate as these do not change within months. Therefore, future work would benefit from using RI-CLPM to examine within-person changes over different intervals (i.e. some studies examine across months and others examine change over many years).

Finally, the current research examines the relationship between personality and income across the working-age population without distinguishing between people in different sectors or stages of career (e.g. whether one is in more junior or senior roles). While this was intentionally done to examine between- and within-person effects across the wider population because such analyses require large sample sizes, it is unclear if these findings would differ across people in specific sectors or at different stages of professional development. Therefore, future work would benefit from more systematically examining such questions across diverse subgroups within the population.

Although the current research raises important considerations, it provides a valuable starting point for future research by demonstrating the importance of examining both within- and between-person effects for the relationship between personality and income.

## 7. Conclusions

Between-person analysis showed that higher incomes were obtained by both men and women who were more extraverted, agreeable, open, and less neurotic. Small within-person effects were also found such that people who became more extraverted over time tended to earn less, while income increases were followed by increases in neuroticism.

## Data Availability

NZAVS data are hosted at the University of Auckland, New Zealand. Data cannot be made available due to ethical restrictions imposed by the University of Auckland Human Participants Ethics Committee. A de-identified dataset is available to appropriately qualified researchers upon request from the last author, any member of the NZAVS advisory board, or the Chair of the University of Auckland Human Participants Ethics Committee. Supplementary material is available online [41].
